# Diagnostic cervical excision in patients with HPV positivity, cytological abnormalities- and preoperative cervical stenosis

**DOI:** 10.1186/s12905-024-03195-9

**Published:** 2024-07-19

**Authors:** Agnieszka Denecke, Duaa Emar, Peter Hillemanns, Dhanya Ramachandran, Bianca Brüning, Matthias Jentschke

**Affiliations:** 1 Department of Obstetrics and Gynaecology, Hannover Medical School, Hannover, Germany; 2 Department of Obstetrics and Gynaecology, Klinikum Wolfsburg, Wolfsburg, Germany; 3 Sociology Department, Leibniz University, Hannover, Germany

**Keywords:** Diagnostic conization, HPV screening, Cervical stenosis

## Abstract

**Aim:**

There is currently no protocol for classifying patients with HPV persistence and preoperative stenosis of the cervical canal. This has a significant impact on cytology results, colposcopy results and the possibility of obtaining reliable cervical histology outcomes. Our analysis clearly shows that colposcopy and cytology underestimate the histological results in patients with limited visibility due to the presence of a type 3 transformation zone (TZ). Our analysis revealed a significant discrepancy between the colposcopy and cytology results and the histological outcomes. Insufficient colposcopy led to the underdiagnosis of dysplastic lesions in patients with a type 3 TZ and cervical stenosis. In the case of repeated cytological abnormalities and inadequate colposcopy examination, it is crucial to perform a diagnostic conization to exclude high-grade dysplastic changes and cervical carcinoma.

**Methods:**

We conducted a retrospective analysis of 1,021 conizations performed in tertiary care hospital in Wolfsburg, Germany between 2014 and 2020. Of these surgical procedures, 89 were diagnostic conizations. In our analysis, we defined diagnostic conization as a procedure performed when there is HPV persistence and repeated cytologic abnormalities in combination with a type 3 TZ, and when it is not possible to retrieve a relevant cervical histology sample.

**Results:**

In this period, 8.7% of all conizations were diagnostic excisions. We found histological abnormalities in 48 of 89 patients (53.9%). The histological examination of the excised cone revealed high-grade cervical intraepithelial neoplasia (CIN/HSIL) in 9 patients (10.1%) and CIN 2+ (HSIL) in 23 out of the 89 patients (25.8%). Two cases of early-stage cervical carcinoma (FIGO IA1 and FIGO IA2) were confirmed (2.3%).

**Conclusion:**

Patients with cervical stenosis, high-risk HPV persistence and repeated cytological abnormalities are at high risk of undetected high-grade cervical dysplasia. Histologic confirmation must be ensured in this patient consultation and this can be achieved by performing diagnostic excisions.

## Introduction

Early detection is the key to successful management and treatment of cervical cancer. The development of several methods in the past decades has been nothing short of remarkable [1]. The most common screen test, as well as the Pap smear, is based mainly on using a brush to remove a small part of the lining tissue and checking it under a microscope to see if there are changes in the cell. This type of test can be used to discover an infection, inflammation, the presence of the HPV virus and cancer. The manual Pap test is highly dependent on the pathologist’s expertise [2]. In recent decades, we have seen the continuous development of cytology as the most widely used detection method. The Pap smear test has undergone several improvements, including the development of liquid-based cytology, the introduction of computer-aided diagnosis (CAD) and, since 2000, machine learning (ML) algorithms and 3D imaging and fluorescence spectrometry, which has improved its accuracy and higher specificity and sensitivity [3].

The development of HPV tests represented another significant advance in the diagnosis of cervical carcinoma. There are several methods for detecting HPV nucleic acids (HPV DNA, HPV oncogene mRNA) that can be used to diagnose HPV infections. These include numerous tests for detecting HPV DNA or HPV oncogene mRNA based on PCR, isothermal nucleic acid amplification or signal amplification [4].

Cytology and histology can be combined with immunocytochemical or immunohistochemical detection of HPV proteins or cellular proteins whose expression is altered by HPV infection (e.g. dual stain p16/Ki-67 staining) and represents an effective triage method for women with LSIL cytology [5].

The German cervical cancer screening protocol has been updated since 2020 to include two pillars for women aged 35 and older: HPV testing and cytology. HPV-positive women must undergo cytological and colposcopy examinations to determine further management [6]. The procedure defined by the German federal joint committee (Gemeinsamer Bundesausschuss, G-BA) is clear: women with high-grade cervical intraepithelial neoplasia /Pap IV and V findings (HSIL) in co-testing (HPV and cytology) screens must be referred to colposcopy within three months. The same applies to women with cytology results of Pap III D2 (HSIL) and Pap III p/e/g (atypical squamous cells-cannot exclude HSIL/ACS-H), irrespective of HPV status. In contrast, women with Pap II p (atypical squamous cells of undetermined significance/ ASC-US), Pap II g/e (atypical glandular cells/ AGC) and Pap III D1 (low-grade squamous intraepithelial lesion/LSIL) findings should be referred within three months only in the case of a positive HPV test. Participants with Pap I findings and a negative HPV test will be invited for subsequent routine screening after three years [7].

It is well established that women with normal cytology results (ASCUS) and long-lasting HPV have an increased risk of developing HSIL. This has been described in numerous studies [8].

The quality of a colposcopy depends on the examiner’s experience. A second important factor is the patient’s pathological anatomical conditions, which often affect the quality of the examination. These include stenosis of the orifice due to atrophy, inflammation, or uterine involution. Colposcopy is a crucial tool in cases of discrepant and unclear findings, and it provides clear indications for surgical therapy in routine medical practice [9]. The key criterion for sufficient performance of colposcopic examinations is the recognition of the squamocolumnar epithelial junction. The assessment of visibility is also an important factor in further patient management and determining the excision type [10]. Visibility is assessed as follows: TZ 1 and 2 are visible, while TZ 3 is not completely visible. Petry et al. definitively demonstrated that colposcopy has a lower specificity in patients with a type 3 TZ or the presence of glandular changes in terms of ACIS. The sensitivity and specificity of colposcopy in the case of a type 3 TZ are unclear [11].

The S3 guidelines for the diagnosis, therapy, and follow-up of patients with cervical carcinoma clearly set out the indications for performing cervical excisional surgery. These guidelines distinguish between diagnostic and allow for the use of different techniques. Conization is the standard procedure for patients with biopsy-proven HSILs [12].

It is widely accepted that in the case of pathologically discrepant findings between conspicuous cytological smears and normal histology, a diagnostic cervical excision is indicated [13].

There is a clear lack of evidence for diagnostic excisions, as well as a record of the indications for diagnostic excisions. There is no consensus or proper guidelines about the treatment of patients with cervical stenosis. In Germany, over 56,000 conizations are performed annually, making it one of the most common surgical procedures. The percentage of diagnostic procedures performed due to discrepancies in cytologic, and colposcopy findings is unknown [14, 15].

The conization procedure is safe and effective with low morbidity and mortality. The long-term negative effects of the procedure described by Arbyn et al. are limited to younger patients and concern about fertility, cervical insufficiency and, ultimately, premature birth. In peri- and postmenopausal patients, intra- and perioperative side effects such as bleeding play a major role [16]. One of the most significant issues for older patients after conization is the risk of unsatisfactory conditions for colposcopy in the future, as well as the possibility of stenosis of the cervix. The surgical technique, depth of surgical excision and postmenopausal status at the time of cervical surgery are the most important risk factors for the development of cervical stenosis. In some cases, it can lead to the development of hematometra, which requires surgical intervention [17]. Santesso et al. conducted a meta-analysis of the side effects of cryotherapy, the loop electrosurgical excision procedure (LEEP) and cold knife conization for the treatment of CIN in more than 2,700 patients from 167 studies [18]. Except for minor bleeding, no serious complications were reported in this patient group. Major infection and pelvic inflammatory disease (PID) have been documented. Intra- and postoperative bleeding is associated with increased rates of type 3 TZ and cervical stenosis [19]. The benefits of diagnostic operative treatment for postmenopausal patients and glandular lesions have been clearly demonstrated in previous studies [20, 21].

It is imperative to evaluate diagnostic interventions in patients with high-risk HPV (HR-HPV) persistence and type 3 TZ. This is because endocervical smear collection is often insufficient and preoperative histological examination is not possible.

## Methods

Our retrospective single-centre evaluation included data from 1,021 conizations performed between 2014 and 2020 in a tertiary care hospital in Germany. We included patients who had undergone diagnostic excision if they met the following criteria: HPV persistence for at least two years, repeated cytological abnormalities (cytology results of Pap III D2 (HSIL) and Pap III p/e/g (atypical squamous cells-cannot exclude HSIL/ACS-H) and Pap III D1 (low-grade squamous intraepithelial lesion/LSIL )and insufficient colposcopy due to a type 3 TZ or cervical stenosis, which did not allow for sufficient preoperative histological examination. Attempts at pre-operative dilatation and endocervical curettage were unsuccessful in all patients. All patients who underwent diagnostic conization in our group were over 40 years old. In younger patients (below 40 years of age), we carried out histological confirmation conservatively with surgical dilatation of the cervix (recanalisation), hysteroscopy and curettage of the endocervix. We used a Kevorkian-Younge curette and applied a corkscrew motion to ensure comprehensive sampling of the full circumference of the canal. The sample should consist of both tissue removed on the curette along with tissue, mucus, and blood collected after curettage with forceps or brush to minimize risk of insufficient sampling. Cervical excision was the recommended procedure when the stenosis involved the external cervical orifice for patients aged older than 40 years according to standards for endocervical curettage [22]. All patients were examined in the colposcopy clinic by colposcopists trained according to the German Society for Colposcopy and Cervical Pathology (AG CPC) before operative treatment was indicated. The HPV tests were carried out using either the Hybrid Capture 2 (HC2, Qiagen, Hidden, Germany) or the COBAS (Roche Diagnostics, Mannheim, Germany) method. All conizations were performed by surgeons with colposcopy experience. Conizations were performed under colposcopy control and exclusively as laser conizations (type 3 excisions) using a CO2 laser to perform deep type 3 resection under general anesthesia. The margins were additionally laser vaporized. An endocervical biopsy was performed as part of the procedure. We obtained informed consent for data analysis. For study purposes, we transferred the Munich nomenclature to the 2015 Bethesda System for Reporting Cervical Cytology, including terminologies [23]. We used the Rio 2011 nomenclature for colposcopy description [24, 25].

All statistical analyses were carried out by an independent statistician. We analyzed the association between HPV infection, cytology, histology, and age in both univariable and multivariable models. All statistical analyses were performed with the validated program SPSS version 28.0 (SPSS Inc., Chicago, IL, USA). Descriptive statistical analysis, Spearman correlations and chi-squared tests were employed. Continuous data are presented as the means ± standard deviations. Categorical data are presented as counts and proportions. A Pearson’s correlation coefficient was calculated for the correlation analysis. A p-value of less than 0.05 was considered to indicate statistical significance.

## Results

We retrospectively analyzed 1.021 conizations performed between 2014 and 2020. Eighty-nine operations were diagnostic excisions and met our inclusion criteria (missing or inconclusive cervical histology results due to cervical stenosis, type 3 TZ, HPV persistence for at least 2 years and repeated cytological abnormalities). All patients in the diagnostic excision group were over 40 years old. The percentage of diagnostic conizations among all cervical excisional surgeries was 8.7%. Three women had previously undergone cervical surgery before receiving diagnostic conization (3.4%). The median age of the patients in the diagnostic group was 48 years (Table 1).

**Table 1:** Characteristics of the patients in diagnostic conisation group (*n*=89), cytological and histological findings

**Age**	**N**	**%**	**Median**
40-50 Y.	36	40.4	54.4 (46-75)
51-60 Y.	31	35.0	
61-70 Y.	18	19.9	
71-80 Y.	4	4.7	
**Operation**	**N**	**%**	
First Conization	86	96.7	
Re - Conization	3	3.3	
**Histology**	**N**	**%**	
CIN 0	37	41.5	
CIN 1	18	20.2	
CIN 1-2	10	11.2	
CIN 2	6	6.7	
CIN 2-3	7	7.9	
CIN 3	9	10.1	
Ca	2	2.2	
**Cytology**	**N**	**%**	
NILM/ Pap I	2	2.2	
NILM/AGC/ASC-US Pap II a, g, k, p	17	19.1	
ASC-H/AGC Pap III p+g	12	13.5	
LSIL/ Pap III D, D1	25	28.1	
HSIL/ Pap III D2	28	31.5	
HSIL/Pap IV a, a-p	5	5.6	

### HR HPV status

All patients tested positive for HR HPV.

Due to the widespread use of HC2 without individual genotyping before the introduction of HPV based screening in Germany in 2020, only 56% of all included patients in our study group had genotyping results. HPV 16 was detected in 29 of our 89 patients (32.6%). 41% of patients with histologically confirmed CIN 3 tested positive for HPV 16, and 25% tested positive for HPV 18 and other HR HPVs (31, 33, 35, 39, 45, 51, 52, 56, 58, 59, 66 and 68). A total of 15.1% of patients had HR HPV persistence for more than 5 years (Table 2). There was no significant association between HPV 16 and CIN 3 (*p* = 0.76) in this small group (Table 3).

**Table 2:** HPV Genotyping distribution

	Study group *N*=89 (100%)	HSIL/CIN 2+ *N*=18 (100%)	HSIL/ CIN 3+ *N*=11
HR HPV	89 (100)	18 (100)	11 (100)
HPV 16,18	29 (32.6)	5 (27.7)	5 (45,4)
HPV OT	4 (4.5)	1 (5.0)	0 (0.0)
Any genotyping information	53 (59)	12(66.6)	6 (54.5)

**Table 3:** Correlations (p)

	**Cytology**	**Histology**
Age	0.198	0.401
HPV 16	0.076	0.135
Major changes	0.485	0.008
Minor changes	0.047	0.055

### Histology

**Fig. 1:**
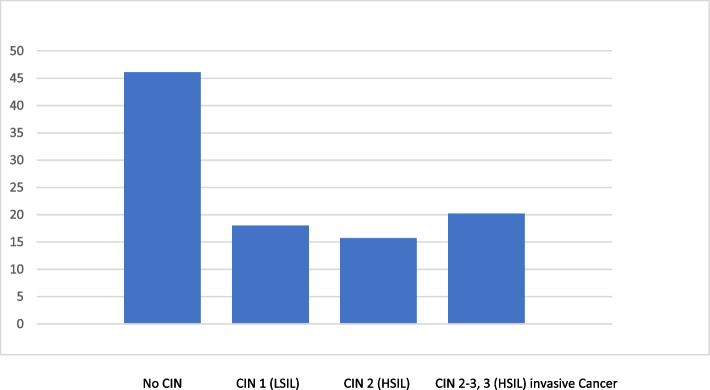
Histologic findings among the patients who underwent diagnostic conizations (*n* = 89)

**Fig. 2:**
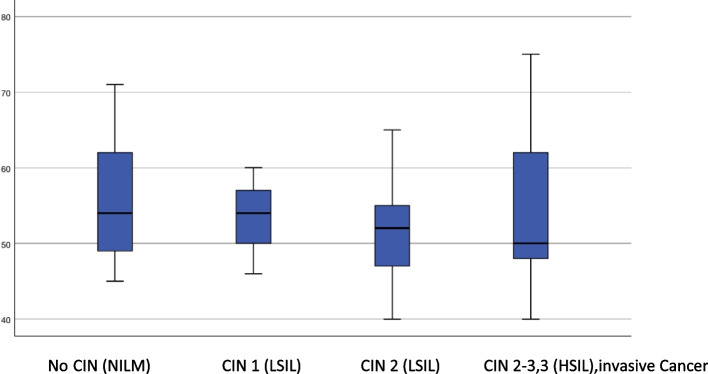
Distribution of histological findings in comparison to the age of the patients in the diagnostic conization group

Among all 89 women who underwent diagnostic conization, 48 patients (53.9%) had histological abnormalities. Histological examination of the tissue revealed CIN 3 (HSIL) in 9 of 88 patients (10.1%). CIN 2+(HSIL) was detected in 23 out of the 89 patients (25.8%). In two patients, early-stage cervical carcinoma was confirmed (squamous cell carcinoma, FIGO IA1 and FIGO IA2 (2.3%)). In 16.8% of the patients, we confirmed LSIL, and in 19% of the patients, we confirmed HSIL (Fig. 1). In two patients, Vaginal Intraepithelial Neoplasia Grade 2–3 (VaIN 2–3) was confirmed by biopsy as an incidental finding during vaginal examination via colposcopy (2.2%). The risk for the detection of severe dysplastic changes was the highest in patients aged 48–58 years (82%). Only 17% of the HSILs were found in patients older than 60 years (Table 4). 42% of CIN 2 (HSIL) cases were detected in patients aged 48–58 years. Even among older female patients (over 60 years old) 50% had CIN 2 (HSIL). 62.5% of LSILs were detected in patients aged 48–58 years. A total of 21% of the patients aged 40–47 years and 17% of the patients aged over 60 years had LSILs (Fig. 2; Table 5, and Table 4).

**Table 4:** Age and histologic finding in diagnostic conization group

	LSIL/CIN1 (*N*=18)	HSIL / CIN 1 2,2 (*N*=16)	HSIL/ CIN 2-3,3, Ca (*N*=18)
40-49 Y.	5	6	8
50-59 Y.	10	8	5
60-69 Y.	2	3	3
>70 Y.	1	0	2

**Table 5:** Cytology and histologic findings in diagnostic conization group

Cytology	*N*=89	LSIL (CIN 1) *N*=18	HSIL (CIN 1-2,2) *N*=16	HSIL (CIN2-3,3) *N*=16	Ca *N*=2
NILM (Pap I +IIa,k) /AGC (Pap II g) /ASC-US (Pap II p)	19	7	2	1	0
ASC-H/ AGC (Pap III p + g)	12	1	1	2	0
LSIL/ (Pap III D, D1)	25	8	4	6	1
HSIL/ Pap III D2	28	2	8	5	1
HSIL/ Pap IV a-p	5	0	1	3	0

During this period, 201 patients (19.7%) over the age of 40 years were treated with laser conization after satisfactory colposcopic examination. Among these 201 patients aged 40 years and older, 38 (18.9%) had no histological abnormalities in the final histology. The LSIL rate was 5.5% (11 patients), which was significantly lower than that in the diagnostic patient population. HSILs were found in 105 patients (59.2%). ACIS was detected in 4 patients (2%). One patient had coexisting high-grade glandular and squamous cervical lesions (0.5%). Fifteen carcinomas were confirmed (7.5%). We did not observe a single cervical adenocarcinoma in the therapeutic conization group.

### Cytology

Most patients (52 out of 89, 58.4%) in the smear group had low grade intraepithelial lesion (LSILs).

Twelve patients (13.5%) were diagnosed with atypical squamous cells cannot exclude HSIL (ASC-H) and atypical glandular cells (AGC endometrial NOS). Twenty patients (22.4%) were negative for intraepithelial lesion or malignancy (NILM), and only 5 (5.6%) were assigned to the HSIL group. We can confirm that there were no cases of carcinoma within this group (Table 5).

In the group of patients with confirmed HSILs after conization, 50% had LSILs (Pap III D1) and HSILs (Pap III D2), and 16.8% had ASC-H and NOS (Pap IIIg/p). Pap IV a-p was found in only 5.6% (HSIL) of the patients (Table 5).

The cytological results from gynecologists in private practices and our colposcopy clinic were found to correlate significantly with our analysis (*p* < 0.01, Table 5).

### Colposcopic examination

Our analysis revealed a clear correlation between minor changes in the cervix and patient age (*p* < 0.001). However, we did not find a significant correlation between major changes in the ectocervix and HSIL findings. In patients who were older and had limited assessment conditions due to a type 3 TZ or cervical stenosis, minor changes indicating dysplastic changes of varying degrees were more likely to occur.

## Discussion

Our analysis focused on the retrospective evaluation of cervical excision for long-term cytological abnormalities, HPV persistence, and preoperative cervical stenosis. Investigators must address the challenges presented by this group of patients. It is crucial to ensure diagnostic certainty, exclude carcinoma and avoid overtreatment. There is a lack of data regarding the percentage of procedures in patients with HPV persistence and cytological abnormalities and a type 3 TZ, for whom histological confirmation is not possible. In our analysis, these operative procedures took place in 8.7% of the cohort, which is lower than that described elsewhere. We were able to show that excisional therapy can be limited if you have strict patient selection criteria. We know from other patient groups that preoperative classification of intrauterine adhesions can be important and have an impact on treatment and prognosis [26]. Unfortunately, this patients group lacks such an important classification system. In our opinion, it is even more important to select patients very carefully for perform excisional diagnostic. In addition to histological clarification, this can lead to new stenosis.

The most important aspect of the study was to determine the percentage of high-grade dysplastic changes (HSILs and carcinomas). Our analysis also aimed to determine the correlation between cytology, colposcopy, and histology results after surgery. Our analysis demonstrated that colposcopy and cytology results underestimated the histology result in patients with limited visibility due to the presence of a type 3 TZ. The rate of severe dysplastic changes in our study was 18%, which is significantly lower than that reported in other similar studies, such as the study by Matthews et al. (75%, [27]). However, we did identify two cases of early curative-stage cervical cancer in our patient group. Table 5 shows that the risk of detecting severe dysplastic changes was highest in patients aged 50–59 years. Our findings demonstrate that colposcopy and cytology results do not significantly correlate with histology results (Table 6). The patients with cytological ASCUS or LSIL can also have high-grade histological findings. Furthermore, we rarely observed major changes in the ectocervix, especially in the older group of patients. Unfortunately, the lack of signs in colposcopy means that further triage methods need to be developed. It is important, especially in younger patients, to open the cervical canal hysteroscopically, including non-surgical (treatment with Laminaria, Misoprostol, Mifepristone or Dinoprostone) and surgical methods (hysteroscopy, dilatation), to ensure adequate smear and histological sampling. A very important advantage of these procedures would be the avoidance of repeated trauma and renewed stenosis of the cervix. Another clear advantage is the absence of general anesthesia, which significantly reduces the range of side effects for patients and ultimately also the costs [28].

**Table 6:**
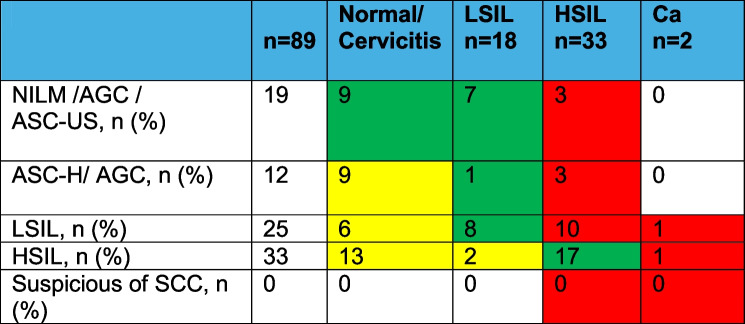
Discrepancy finding graphic (*n*=89)

red: undercall refer to the cytology interpretation in our analysis, green: agreement, yellow: overcall/ minor misfinding

## Results in the context of published literature

There is currently no algorithm or consensus for the further classification and management of patients with HPV persistence and preoperative stenosis of the cervical canal. This has a significant impact on cytological diagnostics and colposcopy. The current literature makes it clear that the results of cytology are not reliable in the case of diagnostic excisions due to HPV persistence and unsatisfactory colposcopy (Table 5). In women over 40 years of age and with low-grade cytology, the HSIL risk is 15% [29]. Katki et al. also reported a 7.4% 5-year risk for HSILs in these patients [30]. Kjaer et al. demonstrated that patients with an equal risk of developing HSILs within 12 years can be stratified according to different HPV genotypes, especially HPV 16 [31]. Another risk factor is patient age. Mittal et al. definitively stated that the highest risk for HSILs is found in women over 50 years of age [32]. It is clear that both cytology and colposcopy are less sensitive for the detection of HSILs in postmenopausal women. This loss of sensitivity can also be explained by the increased proportion of type 2 and 3 TZs. Conization has the consequence of renewed stenosis and the associated difficulty of future colposcopy assessment [33–35].

Targeted use of hysteroscopy and minimally invasive laser surgery, particularly diode laser surgery, could lead to improved diagnosis, outpatient intrauterine synechiolysis and collection of endocervical histology. Currently, the use of new techniques in this area is not widespread due to cost and access [36, 37]. There is also a lack of data on the evidence base for treatment. However, in the future, these advances could lead to a significant improvement in the early detection of cervical cancer and ultimately to a reduction in the need for surgical treatment under general anesthesia and the associated morbidity for patients and costs to the healthcare system.

The management of patients that cannot be assessed accurately by colposcopy and histology due to a type 3 TZ or cervical stenosis is a major challenge in the screening of cervical carcinoma. In these patients, surgical biopsy collection (i.e.cervical excisional surgery) is an option for histological clarification.

Our analysis has several limitations. This was a retrospective case series of patients with unsatisfactory colposcopy results due to cervical stenosis or a type 3 TZ. We found only a small number of diagnostic excisions and a low number of dysplasia, which may have limited the power of our study. This pilot study requires replication in further larger multicentric studies.

### Implications for practice and future research

Our analysis identified the clear benefit of diagnostic excision. Patients who would benefit from diagnostic conization are those whose pathological diagnosis is decided after diagnostic excision and cannot be detected by colposcopy-directed cervical biopsy. It is therefore clear that the risk factors affecting the pathological outcome are not yet fully understood (Table 6).

It is crucial to determine the most appropriate management for HPV-positive women with long-term cytological abnormalities and cervical stenosis. We are confident that HPV genotyping allows the definitive identification of women with significant risk for developing high-grade endocervical lesions in this group.

In conclusion, the analyses of this small series of diagnostic excisions revealed that in the case of repeated cytological abnormalities and inadequate colposcopy examination, histological sampling is essential for excluding high-grade dysplastic changes and cervical carcinoma.

## Data Availability

All data generated or analysed during this study are included in this published article. The database is located in the Department of Gynaecology at Hannover Medical School and Klinikum Wolfsburg. All the data can be obtained from the authors on request.
